# Chromium ion removal from raw water by magnetic iron composites and *Shewanella oneidensis* MR-1

**DOI:** 10.1038/s41598-018-37470-1

**Published:** 2019-03-06

**Authors:** Huiqing Wu, Qingping Wu, Jumei Zhang, Qihui Gu, Linting Wei, Weipeng Guo, Minhong He

**Affiliations:** 1 Guangdong Institute of Microbiology, State Key Laboratory of Applied Microbiology Southern China, 510070, Guangzhou, P.R. China; 2 Guangdong Provincial Key Laboratory of Microbial Culture Collection and Application, Guangdong Open Laboratory of Applied Microbiology, 510070 Guangzhou, P.R. China; 3 Guangzhou Panyu Zhong Village Tap Water Co., Ltd., Guangzhou, Guangdong, 511495 China

## Abstract

In this study, nanoiron active carbon composites (NZVI/GAC) were used to remove chromium ions from raw water. The composites were synthesized from a novel formula of biological activated carbon and characterized by various techniques. The adsorption test data were fit by a pseudo-second-order kinetic model and Langmuir model. The q_e_ and R^2^ values were 187 mg Cr/g and 0.9960, respectively, with 0.2 g/L NZVI/GAC at an initial concentration of 118 mg/L Cr according to the Langmuir isotherm model. Moreover, a Cr^6+^ detoxification reactor was constructed with the magnetic iron composite. The results indicated that the synthesized magnetic iron composite was a significant adsorbent for Cr^6+^ removal from aqueous solutions. The detoxification reactor was able to remove Cr^6+^ from raw water at an initial concentration of 26.5 mg/L within a short time period (3–5 min), with a removal efficiency of up to 99.90% and a treatment capacity of 45.0 mg Cr^6+^/g of adsorbent; the Cr^6+^ concentrations in the outflow met the GB5749–2006 requirements for drinking water. A synergistic effect between NZVI/GAC and a suspension of the bacterium *Shewanella oneidensis* MR-1 was found, showing that this bacterium can be used as a regeneration agent for iron-depleted activated carbon materials.

## Introduction

Water pollution by various toxic contaminants has become one of the most serious problems worldwide^1–5^. Hexavalent chromium (Cr^6+^) is a highly toxic metal and a priority pollutant. The presence of Cr^6+^ in raw water is a potential hazard to aquatic animals and humans and can lead to skin sensitivity and a higher likelihood of genetic defects, including cancer-causing defects^6^. Based on waste management safety standards and the toxicity of Cr^6+^, treatment of this heavy metal in water must be considered. The threshold value for Cr^6+^ in the Hygienic Standard for Drinking Water in the People’s Republic of China GB5746-2006 guidelines is 0.05 mg/L.

Various technologies have been used to treat water and wastewater, including chemical precipitation, ion exchange, adsorption, membrane filtration, coagulation-flocculation, flotation and electrochemical methods^7–9^. The biosorption of heavy metals is highly promising for the removal of toxic metals from industrial waste streams and natural waters^10^. Metal-removal treatment systems that use microorganisms are relatively inexpensive due to the low cost of the sorbent materials and may represent a practical replacement for conventional processes^11–13^. A wide variety of pure and mixed bacterial cultures have been reported to be capable of reducing Cr(VI) under aerobic and/or anaerobic conditions. The Cr^6+^ biosorption properties of algae, bacteria, fungi, and agricultural products and the adsorption properties of non-living substances have been discussed by Bidyut Saha^14^. Among the various treatment options, recent process advancements in nanomaterial sciences have attracted the attention of scientists^15^. Nanoadsorbents not only work rapidly but also have large pollutant-binding capacities. A wide range of nanomaterials have been tested for the removal of inorganic and/or organic pollutants. Many advanced materials such as carbon nanotubes (CNTs) and carbon dots (a photocatalytic material) have been widely used in various fields since their discovery in 1991^16–19^. Given their excellent adsorption, pure and modified CNTs have been successfully used for the purification and enrichment of food, medicines, environmental samples, etc.^20^ Photocatalytic materials for the treatment of pollutants in water are also a popular area of research^21^. Many nanomaterials could eventually represent potent alternatives to conventional treatment methods due to their increased adsorption and/or photocatalytic activity and material specificity, as well as their ability to be chemically regenerated after exhaustion^22^. Such materials include a series of iron-based functional nanomaterials that can use exchange adsorption or chemical reactions to remove heavy metals and toxic organic compounds^23^. Nanoscale metallic iron has been investigated as a new tool for treating contaminated water and soil and has demonstrated versatility for water treatment^24^. The combination of zero-valent nanoiron (NZVI) with supporting materials such as bentonite, activated carbon, bauxite and polystyrene resin reduces the surface energy of the NZVI and enhances its stability without significantly reducing its activity^25–27^. Studies have shown that the integration of biological wastewater treatment processes with advanced nanotechnology results in efficient water purification systems^28,29^. However, all of these nanoparticles can have a major deleterious impact on water quality during treatment processes^30^. Additionally, most applications are not yet market-ready due to technical challenges (e.g., scaling up and system set-up), environmental concerns and cost-effectiveness; therefore, only a few nanosized commercial products are currently available^31,32^.

Because of its high removal efficiency for many pollutants, NZVI can be dispersed and fixed on powdered activated carbon materials to facilitate the construction of a column reactor, thus slowing the decay rate of NZVI and improving its cost-effectiveness while providing advantages of high efficiency, safety and reusability^23,33^. In this study, activated carbon-containing nanoiron particles (NZVI/GAC) and control materials consisting of NZVI nanoparticles (NZVI) were prepared, and the ability of the NZVI/GAC to remove Cr^6+^ when applied alone was evaluated. Furthermore, Cr^6+^ removal reactors were constructed with the NZVI/GAC, and the optimal method for the removal of Cr^6+^ from raw water was studied. Then, the bacterial strain *Shewanella oneidensis* (MR-1) was screened to remove Cr^6+^ from raw water. The potential synergistic relationship between the NZVI/GAC and strain MR-1 was assessed. Finally, to understand the mechanism underlying Cr^6+^ removal, the NZVI/GAC, iron-loaded active carbon composites (GAC-BCS5) and pristine NZVI were characterized by various techniques, such as transmission and scanning electron microscopy (TEM and SEM, respectively), X-ray diffraction (XRD), and Fourier transform infrared spectroscopy (FTIR), and the properties of the synthetic materials, including their Brunauer-Emmett-Teller (BET), surface area (m^2^/g), zeta potential (mV) and total iron content, were evaluated.

## Results

### Sorption isotherms and kinetics of the NZVI/GAC material

**Figure 1:**
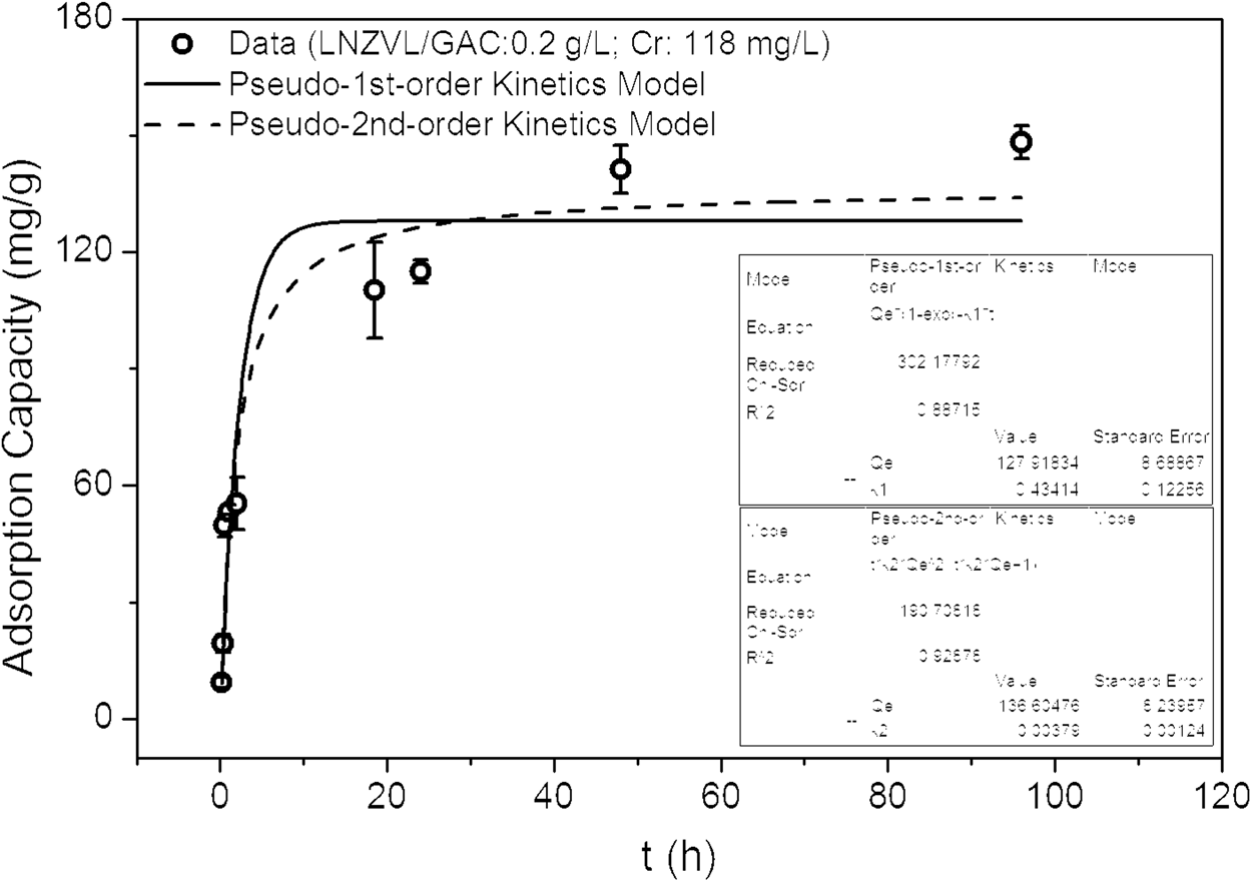
The kinetics of NZVI/GAC (0.2 g/L) and Cr at an initial concentration of 118 mg/L.

**Figure 2:**
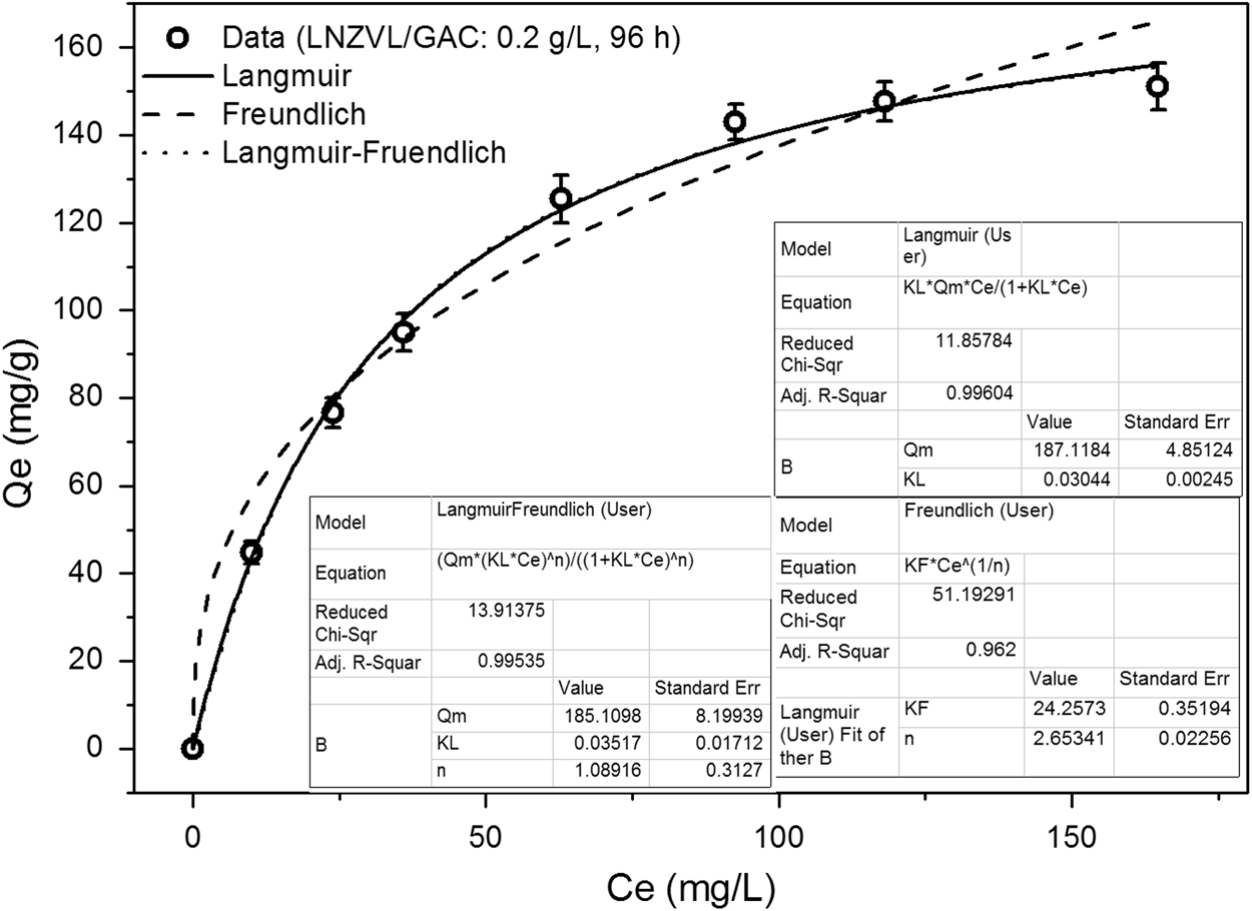
The sorption isotherms of NZVI/GAC (0.2 g/L) and Cr at initial concentrations of 0–175 mg/L.

Figure 1 shows the kinetics of the NZVI/GAC material at a concentration of 0.2 g/L with an initial Cr concentration of 118 mg/L. Figure 2 shows the sorption isotherms for NZVI/GAC at 0.2 g/L with initial Cr concentrations ranging from 0–175 mg/L, and Table 1 presents the parameters of the kinetic and thermodynamic models of the synthetic material.

**Table 1:** Kinetic and isotherm models used to fit the chromium adsorption data for NZVI/GAC.

Model	Parameter	Value
**Kinetic models for 0**.**2 g/L NZVI/GAC with 118 mg/L Cr**		
Pseudo-1^st^-order kinetics model	q_e_ (mgg^−1^)	127.918
	K_1_ (min^−1^)	0.43414
	R^2^	0.88715
	RMSD	15.3306
Pseudo-2^nd^-order kinetics model	q_e_ (mgg^−1^)	136.6047
	K_2_ (min^−1^)	0.00379
	R^2^	0.92878
	RMSD	12.17904
**Isotherm models for 0**.**2 g/L NZVI/GAC with 0–175 mg/L Cr**		
Langmuir	K_L_ (Lmg^−1^)	0.03044
	q_m_ (mgg^−1^)	187.1185
	R^2^	0.9960
	RMSD	2.982177
Freundlich	Kf (L^−1/n^mg^−(1−1/n)^)g^−1^)	24.2574
	N	2.6534
	R^2^	0.962
	RMSD	8.6478
Langmuir-Freundlich	q_e_ (mgg^−1^)	185.1098
	K_L_ (min^−1^)	0.03517
	R^2^	0.9954
	N	0.2577
	RMSD	2.948917

The results (Table 1) showed that the pseudo-second-order kinetic model and the Langmuir or Langmuir-Freundlich isotherm model fit the adsorption data. The q_e_ and R^2^ values for NZVI/GAC were 187 mg Cr/g and 0.9960, respectively, at an initial Cr concentration of 118 mg/L with 0.2 g/L NZVI/GAC for the Langmuir-Freundlich isotherm model (Table 1). Based on the R^2^ and RMSD values shown in Table 1, the pseudo-second-order kinetic model predicts a cooperative adsorption process involving adsorbate-adsorption interactions^32,34^. The adsorption rate constant (K_2_ = 0.00379 min^−1^) indicates that the adsorption rate corresponds to a chemisorption process.

### Stability test of the synthetic materials

**Figure 3:**
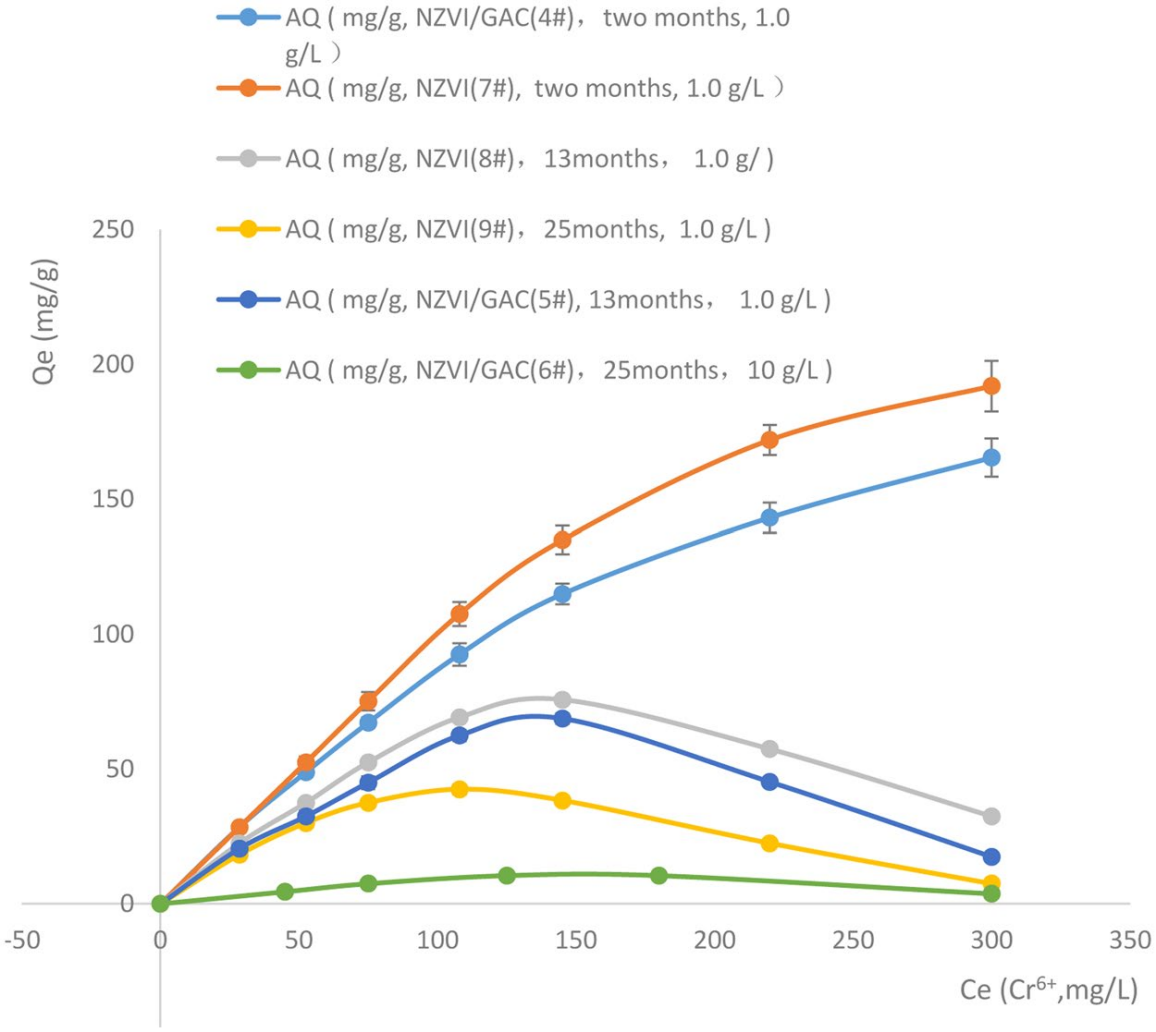
Stability tests of the synthetic materials.

The removal of hexavalent chromium from water by NZVI/GAC with different storage times and NZVI without carrier is shown in Fig. 3.

The results (Fig. 3) show that 1.0 g/L NZVI/GAC and the control NZVI with a storage time of two months (1#, 4#, new material) had maximum adsorption capacities of 165 and 192 mg/g Cr^6+^, respectively. For 1.0 g/L NZVI or NZVI/GAC with a storage time of 12 months (2#, 5#, sub new material) and a range of 0–300 mg/L Cr^6+^, the maximum adsorption capacity was 75.80 and 68.78 mg/g Cr^6+^, respectively, at an initial concentration of 145.15 mg/L Cr^6+^. Comparing the adsorption capacities of several NZVI/GAC after different durations of storage at room temperature (two months, 12 months and 19 months) revealed that the maximum adsorption capacity of NZVI/GAC at a storage time of 12 months was 42% that of the new materials after two months, although high efficacy was retained at a lower initial concentration of Cr^6+^ (<52.5 mg/L). The maximum adsorption capacity of the 19 months samples (3#, 6#, old material) were approximately 10% that of the new material. Comparing the adsorption capacities of the NZVI with storage times of two months, 12 months and 19 months, all at a concentration of 1.0 g/L, showed that the adsorption capacity for Cr^6+^ removal of the NZVI with a storage time of one year was significantly lower than that of the NZVI with a storage time of two months. The maximum adsorption capacity of the NZVI with a storage time of one year was approximately 39.4% that of the new NZVI (at an initial concentration of 145.15 mg/L Cr^6+^), and at a higher concentration of 300 mg/L, the adsorption capacity was approximately 16.9% that of the new material. The maximum adsorption capacity of the NZVI with a storage time of two years was approximately 22.1% that of the new one (with an initial concentration of 145.15 mg/L Cr^6+^). A horizontal comparison showed that the NZVI/GAC with a storage time of 12 months had almost the same adsorption capacity as the NZVI with a storage time of 12 months. These results suggest that the shelf life of synthetic NZVI/GAC is approximately one year.

The performance of NZVI in removing hexavalent chromium from water was slightly superior to that of NZVI/GAC when they were new samples; however, after the two materials were stored in a sealed dry state at room temperature for one year, there was little difference in adsorption capacity for removing hexavalent chromium from water between the two materials.

D. Ribas (2017) reported that dry, bare NZVI particles are highly reactive and are pyrophoric when in contact with air. Current trends in NZVI manufacturing lead to surface passivation of dry NZVI particles with a thin oxide layer, resulting in a decrease in their reactivity^35^. The addition of carriers such as powdered activated carbon to NZVI to produce NZVI/GAC reduces the decrease in surface energy and reactivity of NZVI and increases the yield. Consequently, NZVI/GAC composites can be used in the effective period to improve the cost performance of the materials^23^.

### Growth characteristics and growth curves of *S*. *oneidensis* MR-1

*S*. *oneidensis* MR-1 (MR-1) was grown on Luria broth agar medium (LA) and inoculated into test tubes containing nutrient broth (NB), Luria broth (LB), brain heart infusion broth (NX), MRS broth (MRS) or tryptic soy broth (TSB). The tubes were then incubated at 25–37 °C under aerobic conditions on a shaker with agitation at 150–180 rpm. Growth was determined by observing the development of turbidity in each fermentation broth. The MR-1 strain demonstrated good growth at 30 °C under aerobic conditions in NB, LB, NX, MRS and TSB liquid media.

**Figure 4:**
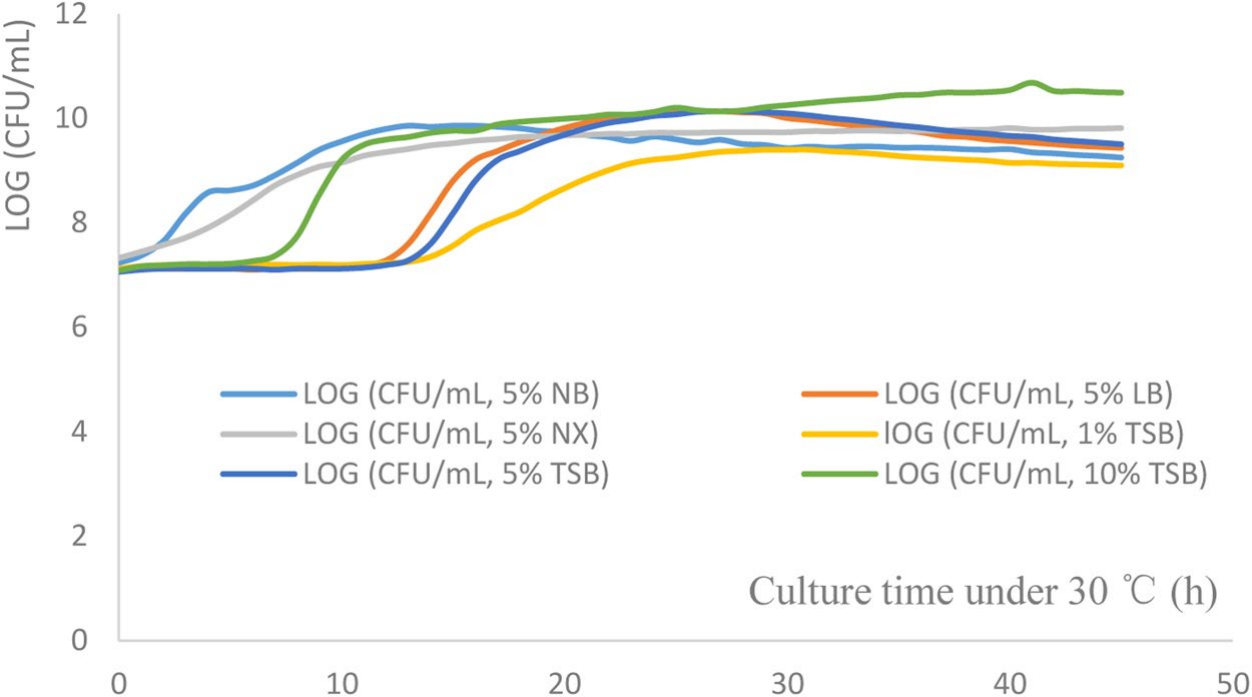
Growth curves of *Shewanella oneidensis* MR-1 in different media.

The growth curves of *S*. *oneidensis* MR-1 cultured in LB, NB, NX, MRS and TSB are shown in Fig. 4.

According to the growth curves of *S*. *oneidensis* MR-1 in different media (Fig. 4), the MR-1 strain had a lag phase of approximately 9–12 h when cultured in LB and TSB with inocula of 1%, 5% and 10% (200 μL of medium was added to the microwell plates and cultured at 30 °C and 150 rpm), and the duration of the lag phase was inversely proportional to the size of the inoculum. The LOG (CFU/mL) value of the MR-1 strain was smaller when it was cultured in TSB at 30 °C for 12 h with an inoculum of 1–5% than when cultured with an inoculum of 10%. The growth curves of the MR-1 strain had shorter lag phases (0.5–1 h) with 5% inocula in NX and NB. Among all tested media, the LOG (CFU/mL) value of MR-1 was greatest in TSB with 10% inoculum after 24 h of growth; therefore, TSB was considered the most suitable medium for cultivation of the MR-1 strain, although all tested media, including NB, NX, LB and TSB, were considered suitable for MR-1 cultivation.

### Cr^6+^ removal by NZVI/GAC and its synergistic relationship with MR-1

**Figure 5:**
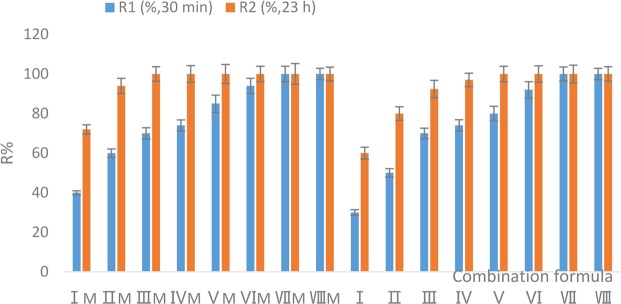
Cr^6+^ removal by NZVI/GAC (2#) with MR-1.

Cr^6+^ removal by the adsorbent formulae I–VIII and IM–VIIIM containing NZVI/GAC (2#) alone or in combination with MR-1 is shown in Fig. 5.

The results showed that 1.6 g/L NZVI/GAC with or without the addition of MR-1 could remove 100% of 37.5 mg/L Cr^6+^ from raw water in 30 min. Strain MR-1 accelerated the removal of Cr^6+^ by NZVI/GAC at the same concentration, and this ability was directly proportional to the MR-1 cell content. Even under large cell concentrations of MR-1, the amount of NZVI/GAC required to remove 100% of the Cr^6+^ did not change; thus, MR-1 clearly had a lower Cr^6+^ removal efficiency than NZVI/GAC(2#).

### Cr^6+^ removal by a NZVI/GAC detoxification reaction column and the synergistic effects of auxiliary water treatment reagents

**Figure 6:**
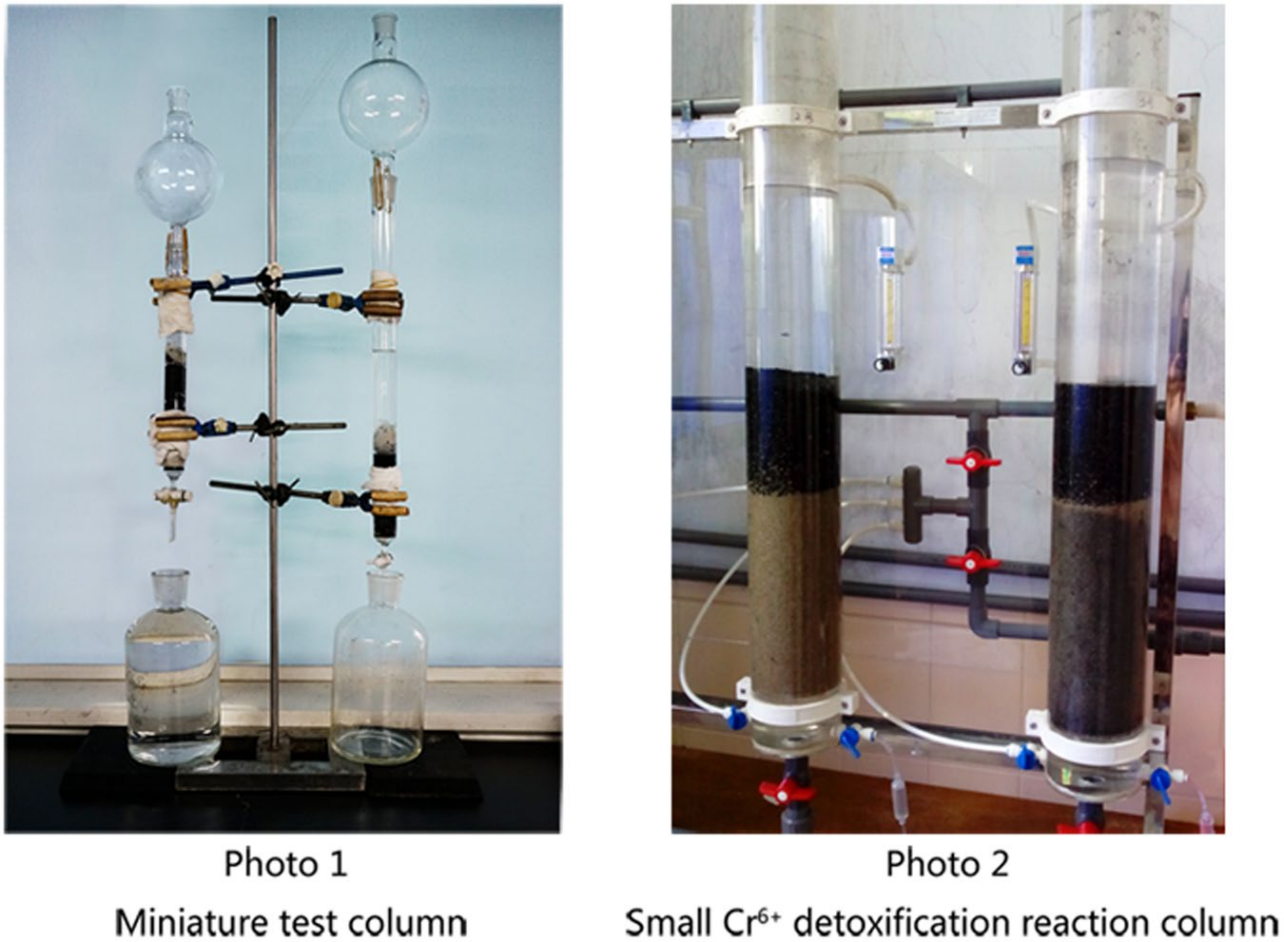
Cr^6+^ detoxification reaction column.

The Cr^6+^ removal and synergistic effects of the NZVI/GAC detoxification reaction column were tested by adding auxiliary water treatment reagents, such as polymeric aluminium chloride (PAC-02), anionic polyacrylamide (PAM) and sodium formate. Photographs of the Cr^6+^ detoxification reaction column are shown in Fig. 6, and the results are presented in Table 2.

**Table 2:** Cr^6+^ removal by the NZVI/GAC detoxification reaction column and synergistic effects of auxiliary water treatment reagents.

Reaction column	Sample group	Initial concentration of the treatment solution (mg/L)	Volume of the inflection point (mL)	Cr content in the outflow water before the turning point (ICP-MS, mg/L)	Remarks
Cr^6+^ detoxification reaction column	Non-additive group	26	800	2.24~4.22	Pure water
	Additive group	26	3705	0.01~0.03	Pure water and auxiliary reagents
Control reaction column	Additive group	26	30*	15.05	Pure water and auxiliary reagents

^*^This was a blank outflow value of the control reaction column.

The results showed that the control reaction column had no effect on improving the inflection point volume, even when water treatment auxiliary reagents were added. By contrast, for the Cr^6+^ detoxification reaction column packed with 0.5 g of NZVI/GAC and 30 g of K-04 (granular activated carbon for pure water sterilization), adding water treatment auxiliary reagents (2 mmol/L sodium formate + 10 mg/L PAC-02 + 0.5 mg/L PAM) increased the inflection point volume of the fixed plate column for treating hexavalent chromium in water and reduced the content of chromium and other cations in the outflow liquid.

### Cr^6+^ removal and regeneration of the Cr^6+^ detoxification reaction column by MR-1

**Figure 7:**
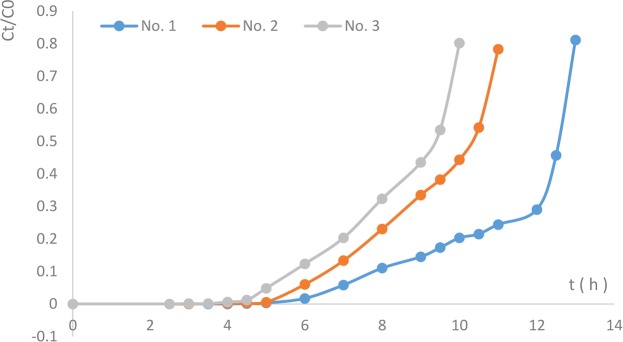
Cr^6+^ removal by the NZVI/GAC (0.5 g) detoxification reaction column with 30 g of K-04 GAC.

The results for Cr^6+^ removal and NZVI/GAC detoxification reaction column regeneration by an MR-1 suspension are shown in Fig. 7.

Under the experimental conditions, the Cr^6+^ detoxification reactor packed with 0.5 g of NZVI/GAC and 30.0 g of K-04 GAC described earlier was able to remove Cr^6+^ at an initial concentration of 26.5 mg/L from raw water within a short time frame (3–5 min) with a removal efficiency of up to 99.90%. Within the inflection point, the Cr^6+^ concentrations in the outflow met the GB5749–2006 requirements for drinking water.

The detoxification reaction column had breakthrough adsorption quantities of 45.0, 42.3 and 35.0 mg of Cr^6+^/g NZVI/GAC, corresponding to saturated adsorption capacities of 154.6, 133.3 and 123.75 mg of Cr^6+^/g NZVI/GAC for the first, second and third column operations, respectively. The Cr^6+^ removal rates at the saturation point of the column were 81.1%, 78.3% and 80.2% for the three operations, respectively, when the column was used to treat 26.5 mg/L Cr^6+^ in raw water. The adsorption amounts at the inflection point and the saturation point of the detoxification reaction column for the third operation were 77% and 80% of those of the first operation, respectively.

### Characterization

#### Electron microscopic analysis of NZVI, GAC-BCS5 and NZVI/GAC

**Figure 8:**
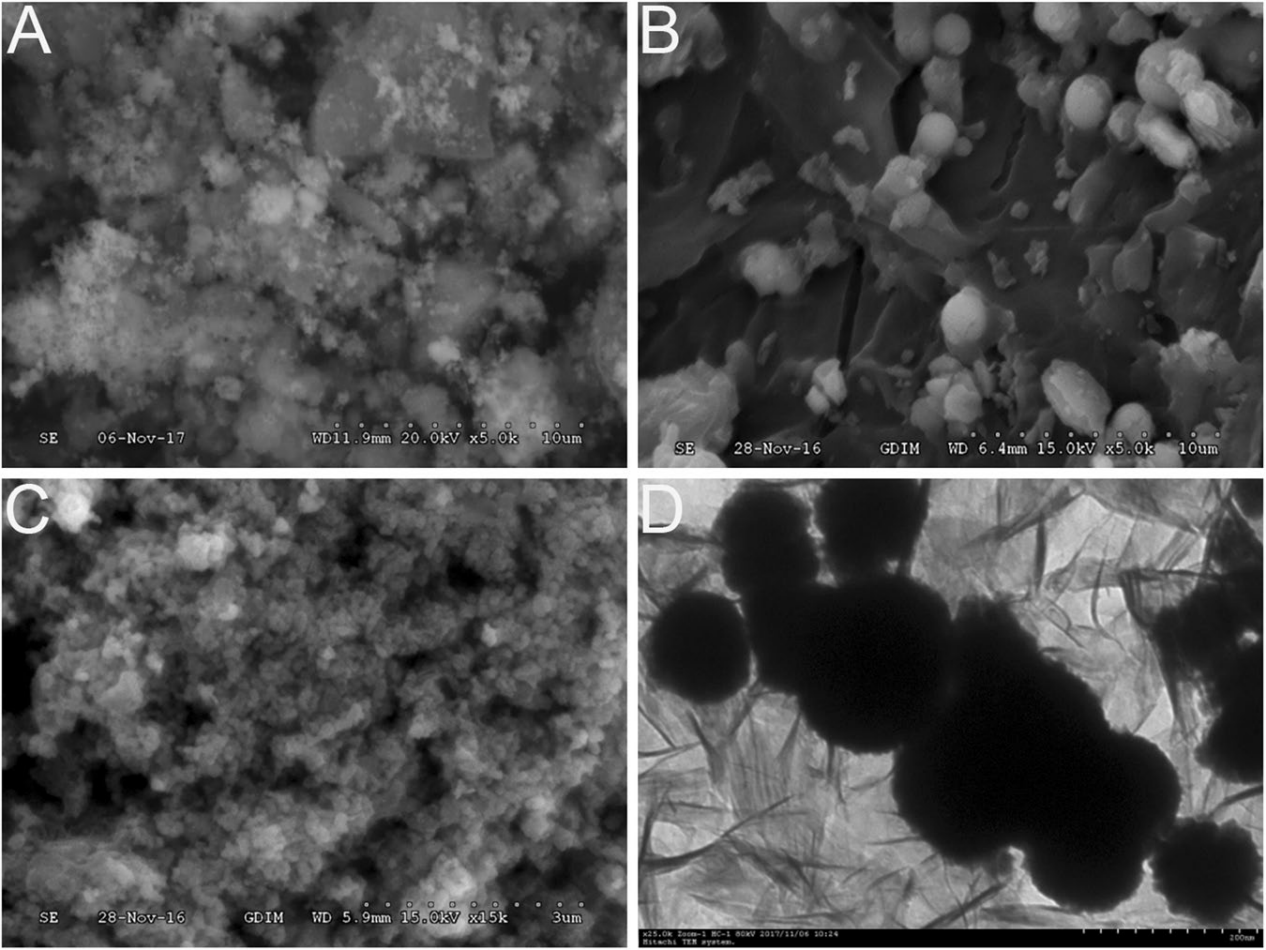
(**A,B,C,D**) Representative SEM images of NZVI/GAC (1#), GAC-BCS5, and NZVI (4#) and a TEM image of NZVI/GAC (1#).

**Figure 9:**
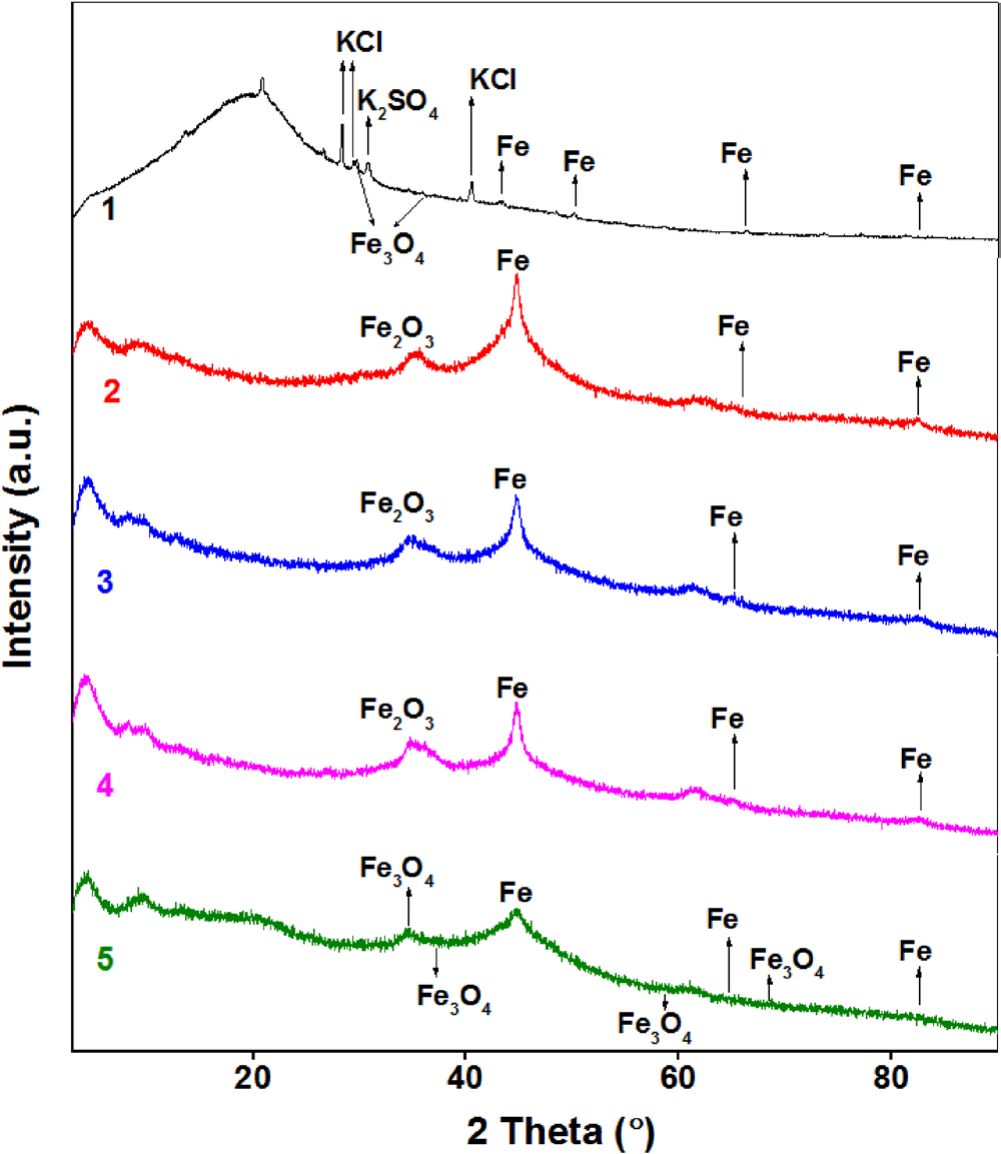
XRD patterns for GAC, NZVI and NZVI-GACs.1: GAC-BCS5; 2: NZVI stored for two months; 3: NZVI stored for one year; 4: NZVI stored for 19 months; 5: NZVI/GAC stored for two months.

TEM and SEM were performed with synthetic nanoscale iron particles (NZVI/GAC), iron-loaded active carbon composites (GAC-BCS5) and pristine NZVI. Figure 8(A–D) shows representative SEM images of NZVI/GAC, GAC-BCS5 and NZVI and a TEM image of NZVI/GAC. Figure 8(A) shows a representative SEM image of NZVI/GAC, which reveals many large floc particles with diameters of 5–15 μm on the surface. As shown in Fig. 8(B), the GAC-BCS5 used to synthesize the nanoscale iron contained a small amount of particulate matter and a large amount of carbon; the fluffy relaxation particles with diameters of 0.15–0.3 μm vary more than the particles on the synthesized NZVI/GAC. Most of the iron nanoparticles of the NZVI/GAC have larger pore sizes than the GACs, suggesting that they are located on the outer walls of the GACs. Unsupported NZVI formed large aggregates, leading to large, chain-like iron nanoparticles, as shown in Fig. 8(C). In the TEM images of NZVI/GAC shown in Fig. 8(D), the dark spots distributed on the GAC support surface are stabilized iron nanoparticles with diameters of 100 nm, and many smaller particles with diameters of approximately 1 nm are attached to the NZVI/GAC, which may be a feature of magnetic materials. The TEM results were consistent with those obtained via XRD (Fig. 9) and N_2_ adsorption-desorption (Table 3).

**Table 3:** Textural properties of NZVI and NZVI/GAC.

No.	S_bet_ (m^2^/g)	Zeta potential (mV)	Total Fe content (mg/kg)
4#	51.123	−7.19	545696.92
5#	23.903	−13.3	576409.50
1#	21.969	−6.81	327316.60

#### XRD

The crystalline properties and composites of GAC BcS5, NZVI/GAC and NZVI were studied using XRD methods. Figure 9 shows the XRD patterns for GAC-BCS5, NZVI/GAC (1#) and three unsupported NZVI (4#, 5#, 6#, corresponding to samples stored for two months, 12 months, and 19 months). Based on comparisons with a database of XRD samples, the results suggested that the crystalline material in GAC-BCS5 contained Fe, Fe_3_O_4_, KCl and K_2_SO_4_, the three unsupported NZVI contained Fe and Fe_2_O_3_, and the NZVI/GAC contained Fe and Fe_3_O_4_. Faint signals indicating the presence of unknown substances in the GAC-BCS5, NZVI/GAC and NZVI were also detected. According to the XRD results, a reflex locus of 2θ reflected the crystalline properties of these five samples. The XRD pattern for the GAC-BCS5 composites showed distinct peaks at approximately 2θ = 43.199, 50.16, and 73.69, corresponding to Fe. According to JCPDS card No. 03-065-4150, 2θ = 29.687 corresponds to Fe_3_O_4_ (JCPDS card No. 01-089-0951); 2θ = 28.321, 29.687, 40.492, 58.54, 66.31 and 73.69 correspond to K_2_SO_4_ (JCPDS card No. 00-005-0613); 2θ = 28.321, 40.492, 58.54, 66.31, and 73.69 correspond to KCL (JCPDS card No. 01-075-0296); 2θ = 13.539, 20.785, 26.581, 29.340, 77.067 and 81.24 are unknown. For NZVI stored for one year, the reflex loci of 35.45, 44.623, 62.03 and 82.51 (JCPDS card No. 01-073-0603) correspond to Fe_2_O_3_, and the reflex loci of 44.623, 62.03 and 82.51 (JCPDS card No. 03-065-4899) correspond to Fe. The XRD pattern for NZVI stored for two months showed distinct peaks at approximately 2θ = 35.17, 44.63, 61.19 and 82.4, corresponding to Fe_2_O_3_ (JCPDS card No. 01-073-0603), and at 2θ = 44.63, 61.19 and 82.4, corresponding to Fe (JCPDS card No. 03-065-4899). The XRD pattern for NZVI stored for19 months showed distinct peaks at approximately 2θ = 35.28, 44.60, 61.27 and 82.82 (JCPDS card No. 01-073-0603), corresponding to Fe_2_O_3_, and peaks at 2θ = 44.60, 61.27 and 82.82, corresponding to Fe (JCPDS card No. 03-065-4899). The XRD pattern of NZVI/GAC showed distinct peaks at approximately 2θ = 35.00, 45.40 and 80.9, which correspond to Fe_3_O_4_ based on a comparison with JCPDS card No. 00-028-0491; peaks at 2θ = 45.40 and 80.9, which correspond to Fe (JCPDS card No. 03-065-4899); and an unknown peak at 2θ = 20.7. Overall, the XRD analysis showed that NZVI/GAC were a magnetic-bearing nanoiron-activated carbon material, while the control materials were nanoscale iron particles containing no magnetic materials.

#### FTIR spectra

**Figure 10:**
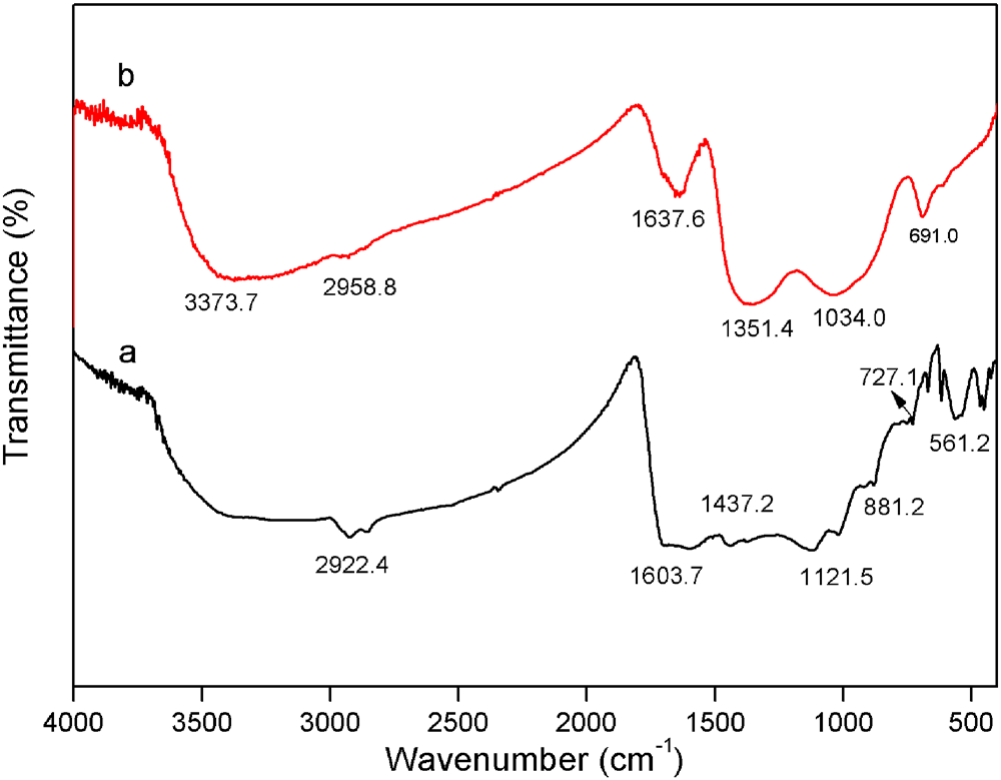
FTIR spectroscopy of a: GAC-BCS5, 100 mesh, and b: NZVI/GAC, 100 mesh.

The FTIR spectra of GAC-BCS5 and NZVI/GAC stored for two months were assessed in the range of 400–4000 cm^−1^ (Fig. 10). Typically, a broad band at approximately 3373.7 cm^−1^ corresponding to an OH group was observed, indicating the presence of hydroxyl groups on the surface of NZVI/GAC^36^. The bands at approximately 2922.4 cm^−1^ and 2958.8 cm^−1^ were assigned to the −CH_2_ and −CH_3_ groups of long-chain aliphatic components, respectively^37^. The characteristic peaks at approximately 1637.6 cm^−1^ and 691.0 cm^−1^ were mainly related to the C=O stretching vibrations of esters and OH groups, respectively^38^. The peak at approximately 1351.4 cm^−1^ was related to −COO groups, and the band near 1100 cm^−1^ was assigned to CO bending vibrations^33^. The weak band at approximately 881.2 cm^−1^ was due to the −CH group of furan. In short, the surfaces of the two types of biochar contained several hydroxyl and carboxyl groups, and modification had little impact on the surface functional groups of these biochars, consistent with the findings of Haoran Dong^23^.

### Determination of the properties of the synthetic materials

The properties of the synthetic materials NZVI/GAC and unsupported NZVI, including their BET surface areas (m^2^/g), zeta potentials (mV) and total iron contents, are listed in Table 3. The BET surface areas of NZVI/GAC stored for two months (1#), unsupported NZVI stored for one year (5#) and NZVI stored for two months (4#) were 21.969, 23.903 and 51.123 m^2^/g, respectively. The observed specific surface area of NZVI/GAC (1#) was lower than that of NZVI(4#,5#) due to the surface coverage of GAC-BCS5 with iron nanoparticles and other particles.

Crystallite sizes were determined using Scherrer’s formula, D = 0.9λ/βcosθ, where λ is the wavelength of the incident X-ray (λ = 1.54056 A), β is the full width at half maximum (FWHM) value of the XRD diffraction lines, and θ is the angle of diffraction, which is half of the diffraction angle of 2θ^39^. The particle size was determined as the average size of the peaks for Fe_3_O_4_ and Fe. Based on this method, the average particle sizes of the Fe_3_O_4_ and Fe deposited on NZVI/GAC (1#, the new material) were approximately 1.4 nm and 7.145 nm, respectively.

According to the results, the NZVI (4#, the sub new material) had a good zeta potential (mV); however, a substantial reduction in the zeta potential was observed after one year of storage under sealed, dry and dark conditions, according to the comparison of NZVI 5# (the sub new material) and NZVI 4# (the new material). The reduction potential and total iron content of NZVI/GAC were lower than those of NZVI, but little difference in surface area was found between the NZVI and NZVI/GAC, which may be related to the screening of the sample with 100 mesh.

## Discussion

The performance of a material in removing hexavalent chromium ions from water is related to its microstructure, including its composition, reactive groups, particle size, and specific surface area, as well as its synthetic GACs and iron-loaded components. NZVI/GAC with high reactivity were synthesized from GACs, which was prepared from a biomass mixture via the modification of specific microbial fermentation products and anaerobic carbonization at high temperature (300 °C). This special activated carbon preparation from fermented biological substrates was used as a carrier for nanoiron, and the NZVI/GAC product exhibited high yield, good dispersity, strong vitality, magnetic adsorption properties and good storage performance. Compared with other supporting materials, such as carbon quantum dots and high-purity graphite and graphene oxide, NZVI/GAC have better cost performance^40,41^(S1). Sources of modified activated carbon are very rich, and the preparation of activated carbon via biological modification combined with nanoiron technology represents a new process for the efficient application of biomass materials for drinking water purification. Like other commercial activated carbon products modified by nanomaterials^42^, the tested material had a strong ability to remove Cr^6+^ from raw water. Additionally, the active carbon loaded with nanoiron was characterized by XRD, which revealed that the material contained both zero-valent iron and Fe_3_O_4_, a magnetic iron-loaded activated carbon composite that has demonstrated good removal capabilities for other heavy metals, such as Cd^2+^, Sb^5+^, Cu^2+^, Zn^2+^ (unpublished data). Such activated carbon materials may also be good treatment agents for the removal of some chlorinated disinfection by-products and environmental endocrine disruptors (EDCS), but further research is required to make this determination^43^.

The Cr^6+^ removal potential of the tested detoxification reactor was related to the reduction abilities of the column materials and their synergistic effects, the flow rate and initial concentration of the Cr^6+^ solution to be treated, and the regeneration ability of the reactor.

**Figure 11:**
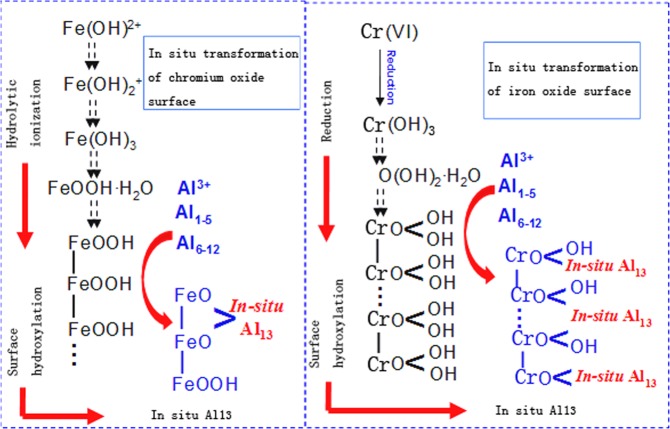
Possible mechanism of the formation and removal of iron and chromium oxides.

The working mechanism of NZVI/GAC for removing hexavalent chromium from raw water in column reactors is as follows (Fig. 11); this process can be expressed by Eqs (1)–(6). When the hexavalent chromium solution supplied by the source water and auxiliaries flows through the reaction column composed of NZVI/GAC and K-04 GAC, the hexavalent chromium is first reduced to trivalent chromium by the NZVI in the system^44^. Then, ferric and trivalent chromium oxides are produced in the process. The sodium formate in the system could be used as an electron donor for the reduction of the high-valent iron^45^. *In situ*, trivalent iron is formed by hydroxylation, forming Fe (OH)_3_. The iron oxidation and Cr (VI) reduction reactions are favoured at low pH^46^. The formation of a pH-lowering microenvironment on the colloidal surface (Pac-02) and a proton-transferring microinterface process can control the formation of intermediates with different degrees of polymerization of Al through Al(OH)_3_ + H^+^, and the cationic polymer polyacrylamide (PAM) could be used as a coagulant aid^47^. The D-101 macroporous adsorbent resin used as a supporting material in the reactor is a styrene-type non-polar copolymer with a wide range of applications. It has a strong general adsorption capacity for organic compounds with no or weak polarity. Hence, before the inflection point, the reaction system has enough activity to reduce the hexavalent chromium in the water to trivalent chromium. The effluent water is clear and transparent, and the oxides of chromium and iron and most organic compounds are removed. After the inflection point, the reductive power of the system is not sufficient to reduce all the hexavalent chromium in the water, and part of the unreduced hexavalent chromium flows out of the effluent until the reaction activity of the system disappears. At this time, the reaction column is saturated and has no further reductive power for hexavalent chromium.

1 $$2{{\rm{Fe}}}^{0}+4{{\rm{H}}}^{+}+{{\rm{O}}}_{2}\to 2{{\rm{Fe}}}^{2+}+2{{\rm{H}}}_{2}{\rm{O}}$$

2 $${{\rm{Fe}}}^{0}+2{{\rm{H}}}_{2}{\rm{O}}\to {{\rm{Fe}}}^{2+}+{{\rm{H}}}_{2}+2{\rm{OH}}-$$

3 $${{\rm{Fe}}}^{2+}+{{\rm{H}}}_{2}{{\rm{CrO}}}_{4}+{{\rm{H}}}^{+}\to {{\rm{Fe}}}^{3+}+{{\rm{H}}}_{3}{{\rm{CrO}}}_{4}$$

4 $${{\rm{Fe}}}^{2+}+{{\rm{H}}}_{3}{{\rm{CrO}}}_{4}+{{\rm{H}}}^{+}\to {{\rm{Fe}}}^{3+}+{{\rm{H}}}_{4}{{\rm{CrO}}}_{4}$$

5 $${{\rm{Fe}}}^{2+}+{{\rm{H}}}_{4}{{\rm{CrO}}}_{4}+{{\rm{H}}}^{+}\to {{\rm{Fe}}}^{3+}+{\rm{Cr}}{({\rm{OH}})}_{3}+{{\rm{H}}}_{2}{\rm{O}}$$

6 $${{\rm{Fe}}}^{3+}+3{{\rm{H}}}_{2}{\rm{O}}\to {\rm{Fe}}{({\rm{OH}})}_{3}+3{{\rm{H}}}^{+}$$

Heavy metals and metalloids can be effectively removed from metal-laden biosorbents using dilute acids (e.g., HCl, HNO_3_, and H_2_SO_4_)^48^. In this study, 0.01 mmol/L hydrochloric acid was used to elute the columns after the adsorption of metal ions, and a MR-1 bacterial solution was then used to regenerate the iron-loaded activated carbon materials. The synergistic action of the metal-reducing bacterium *Shewanella oneidensis* MR-1 and the iron-loaded activated carbon may be related to the performance of the MR-1 bacteria. Because *S*. *oneidensis* MR-1 is a Gram-negative proteobacterium, it inhabits a wide variety of niches in nature and has a characteristic ability to reduce a broad spectrum of electron acceptors, such as metals, nitrates, thiosulfates, dimethyl sulfoxide, trimethylamine N-oxide, fumarates and azo dyes, in addition to O_2_^49^. Extracellular electron transfer (EET) is a key feature of *S*. *oneidensis* MR-1. C-type cytochromes can be used as carriers to transfer electrons and play important roles in EET processes. The genome of *S*. *oneidensis* MR-1 encodes three terminal oxidases: a bd-type quinol oxidase plus two haem-copper oxidases, a cytochrome c oxidase (genes SO4606–SO4609) and a cbb3-type oxidase (genes SO2361–SO2364)^50,51^.

## Conclusion

This study provides a method for the removal of Cr^6+^ from raw water using a synthetic activated carbon-containing magnetic nanoiron (NZVI/GAC) with high reactivity. The q_e_ and R^2^ values were 187 mg Crss/g and 0.9960, respectively, for 0.2 g/L NZVI/GAC at an initial concentration of 118 mg/L Cr according to the Langmuir isotherm model. The adsorption test results showed that a pseudo-second-order kinetic model and Langmuir model fit the adsorption data. The adsorption rate constant (K_2_ = 0.00379 min^−1^) indicates that the adsorption rate corresponds to a chemisorption process.

The Cr^6+^ detoxification reactor constructed with NZVI/GAC (new material) was able to remove up to 99.90% of Cr^6+^ ions from raw water with an initial concentration of 26.5 mg/L within a short time period (3~5 min), corresponding to a treatment capacity of 45 mg Cr^6+^/g of NZVI/GAC. The Cr^6+^ concentrations in the outflow met the requirements of the GB5749-2006 drinking water standard. When the concentration of Cr^6+^ in the effluent was 81.1% of the initial concentration in the influent, a treatment capacity of 154.6 mg Cr^6+^/g of NZVI/GAC adsorbent was obtained. The synergistic effect between NZVI/GAC and the MR-1 bacterial suspension was harnessed to further improve the Cr^6+^ removal. For these synthetic materials, the MR-1 bacterial suspension enabled the regeneration of iron-depleted activated carbon. The heavy metal detoxification reactor built using the nanomagnetic iron active carbon material had high efficiency, and the effluent at the inflection point met the requirements of the national standard. This reactor, which can be used to treat hexavalent chromium and other heavy metal-polluted water, is easy to prepare, renewable, simple to expand and inexpensive.

## Materials and Methods

### Strains

*Shewanella oneidensis* MR-1 (MR-1) is one of the most well-characterized strains of bacteria and was originally isolated from Oneida Lake, NY, USA^52^. For this study, MR-1 was purchased from the Marine Culture Collection of China (MCCC), which is supported by the Guangdong Institute of Soil Ecology^53^. The metal-tolerant bacterium *Bacillus cereus* S5 was isolated from soil at the Liuyang Xiang River chemical plant in Changsha^54^. The culture media used in this study, including LB, NB, TSB, TSA, NX and MRS media, were purchased from Guangdong Huankai Microbial Technology Co., Ltd., Guangzhou, China.

The Cr^6+^ solution was prepared from potassium dichromate (AR). All reagents, including potassium dichromate, sodium formate, and FeSO_4_·7H_2_O, were purchased from Guangzhou Shuo Heng Chemical Reagent Co., Ltd, GuangZhou, China. PAM, anionic number ≥30000, was purchased from Guangzhou Fang Bo Environmental Protection Technology Co., Ltd. White PAC-02 (drinking water grade) and Al_2_O_3_ (≥29%) were purchased from Guangzhou Jin Xin Chemical Co., Ltd. GuangZhou, China.

All solutions were prepared using Milli-Q deoxygenated ultrapure water (18 MV cm, Easy Pure II RF/UV, USA).

The support materials for the construction of the hexavalent chromium reaction column were as follows: (1) K-04-activated carbon from coconut husk (Hainan Star Activated Carbon Co., Ltd., model K-04,10–20 mesh), with a filling density of 0.40~0.55 g/mL, a specific surface area of more than 11000 m^2^/g, and more than 90% of particles with sizes between 10 and ~28 mesh; (2) the macroporous resin D 101; (3) medical skimmed cotton purchased from Guangzhou Shuo Heng Chemical Reagent Co., Ltd.; (4) water of different purities, including pure water and ultrapure water prepared in the laboratory and raw water from the Guangzhou Panyu Tap Water Company’s Village Tap Water plant.

### Preparation of specially formulated biological activated carbon (GAC)

The GAC preparation process mainly included solid-state fermentation of a mixture of biomaterials, followed by low-temperature drying, crushing and anaerobic carbonization, crushing, preservation, etc.

In this study, the microbial strain BCS5, a strain of *B*. *cereus* isolated from heavy metal-polluted soil that is tolerant to heavy metals such as cadmium and antimony, was used to prepare the biological activated carbon.

A solid-medium formulation of biomass materials was developed. The biomass was a mixture of corn, wheat bran and soybean meal at a ratio of 3:4:2 by weight. Nutritive salts, including 0.1% MgSO_4_·7H_2_O, 0.1% peptone, 0.1% KH_2_PO_4_, 0.2% K_2_HPO_4_·3H_2_O, 0.1% FeSO_4_ 7H_2_O, and 0.1% CaCO_3_, were calculated according to their total amount and were dissolved in 400 mL of pure water per 500 g of dry biomass mixture. The manufacturing process for the specially formulated biological activated carbon was as follows: after mixing the biomass with a nutrient solution and sterilizing at 121 °C for 20 min, 10–30% (v/w) BCS5 in TSB (37 °C, 180 rpm agitation, 1 d) was inoculated into cooled solid medium and fermented for 3 d at 37 °C. The fermented medium was dried at 60–80 °C, crushed and sieved through 20 mesh, and anaerobically carbonized at 300 °C for 2–3 h in a muffle furnace with a 70% maximum heating rate. The obtained activated carbon powder was gradually cooled to room temperature. After crushing and screening, finer activated carbon powders corresponding to mesh size of 100 were obtained. These finer activated carbon powders were vacuum-sealed and stored under the protection of inert argon gas or argon with nitrogen. The biological activated carbon obtained from the fermentation of a biomass mixture as described above was referred to as GAC-BCS5AM. The final mass of the specially formulated powder obtained from an initial dry biomass mixture of 500 g via fermentation, drying (50–90 °C) and anaerobic carbonization at 300 °C for 2–3 h in a muffle furnace with a 70% maximum heating rate was approximately 180 g (mean value, SD: 5%).

### Preparation of the specially formulated NZVI/GAC

**Figure 12:**
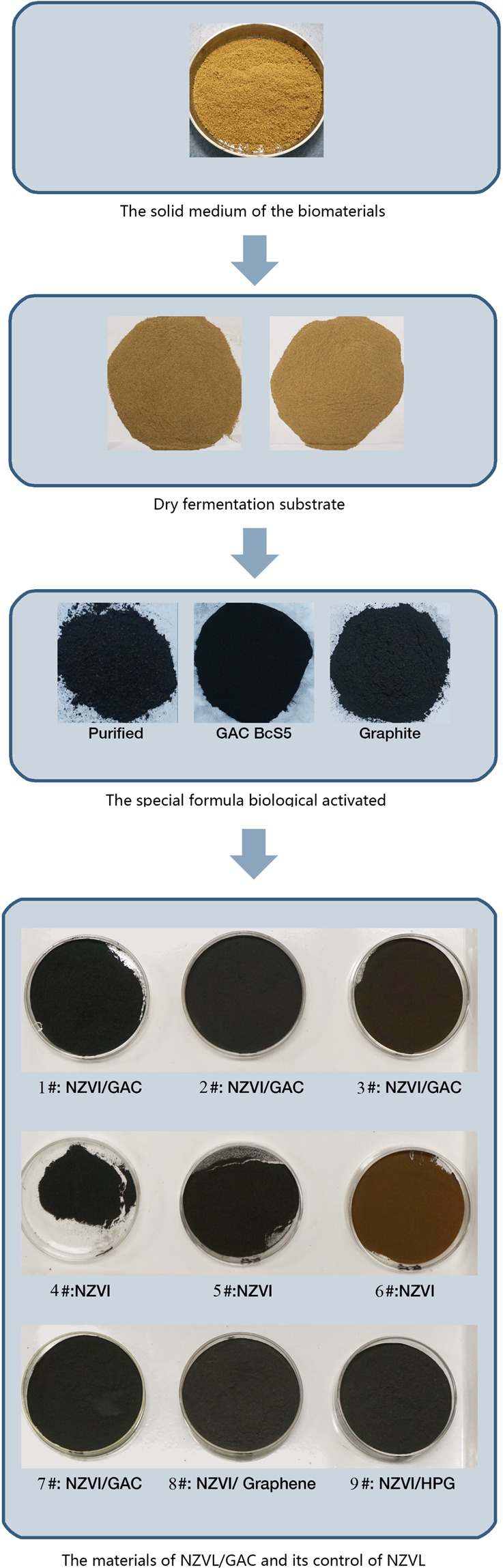
Diagram of NZVI/GAC preparation.

A liquid-phase reduction method was employed for the synthesis of NZVI/GAC^55,56^, and a typical preparation diagram is depicted in Fig. 12. For comparison, unsupported pristine NZVI were synthesized in a similar manner without the addition of GAC, according to Eq. (7). The prepared material was dried under a vacuum in a drying oven and stored in vials with a vacuum seal under the protection of inert argon gas for subsequent experiments.

7 $${{\rm{Fe}}}^{2+}+2{{{\rm{BH}}}_{4}}^{-}+6{{\rm{H}}}_{2}{\rm{O}}\to {\rm{Fe}}0\downarrow +2{\rm{B}}{({\rm{OH}})}_{3}+7{{\rm{H}}}_{2}\uparrow $$

NZVI/GAC were prepared in this study via improved methods. Preparation began with 10 g of 100-mesh GAC and 1.6 mol/L FeSO_4_·7H_2_O in 100 mL of 1% PEG-6000 with degassed water treated with ultrasound at 80 kHz for 120 min. Subsequently, a freshly prepared NaBH_4_ solution with 30% (v/v) degassed water/absolute ethanol (moles of NaBH_4_:Fe^2+^ = 2:1) was added to the mixture at a rate of 50–60 drops per minute. After delivering all the NaBH_4_ solution (dissolved in degassed water), the mixture was sealed for 2 h of quiescent reaction. Next, the prepared composites were separated from the solution by centrifugation, followed by rinsing with deionized water and centrifugation twice. The composites were then dried under vacuum in a drying oven at 80–90 °C and stored in vials for subsequent experiments under the protection of inert argon gas. A mean of 25.0 g of NZVI/GAC (SD: 5%) was obtained from 10 g of GAC-BCS5AM (100 mesh).

To prepare samples of NZVI, 41.7 g of FeSO_4_·7H_2_O (AR, 0.15 mol) was dissolved in 100 mL of 1% PEG-6000 in degassed water. After stirring evenly, 11.36 g of NaBH_4_ (0.3 mol) was dissolved in 100 mL of 30% (v/v) degassed water/absolute ethanol, and this freshly prepared NaBH_4_ solution was added to the mixture at a rate of 50–60 drops per minute, followed by sealing for 2 h of quiescent reaction. The remaining steps were the same as those for the NZVI/GAC preparation.

Sample numbers in this paper: 1#, NZVI/GAC (201804050302); 2#, NZVI/GAC (20170602B5); 3#, NZVI/GAC (201611050302); 4#, NZVI (201804050301); 5#, NZVI (20170602C2); 6#, NZVI (201611050301); 7#, NZVI/GAC (2018091802); 8#, NZVI/graphene (2018091804); 9#, NZVI/HPG (2018091806). Numbers 1#–6# were presented as samples in this paper, and numbers 7#–9# are samples presented in the supplementary material. All studies were conducted within the validity period (not exceeding one year, usually within two months), except for the validity test samples.

### Sorption isotherms and kinetics of NZVI/GAC

Cr^6+^ removal by NZVI and NZVI/GAC with adsorbent concentrations of 0.2–10 g/L was tested in this study. Cr^6+^-polluted water was prepared with pure water and potassium dichromate at initial concentrations of 0–300 mg/L Cr^6+^. The processing system used 50 mL of Cr^6+^ solution and 10–500 mg of NZVI/GAC, which were mixed and reacted for 24 h at room temperature. Samples taken at set time intervals were centrifuged at 12600 rpm for 2 min and diluted to the appropriate range for detection. The Cr content in the supernatants was measured via ICP-MS (Agilent 1260–7700e; Agilent Technologies Co. Ltd, USA). The residual concentration of Cr^6+^ in the solution was analysed with a heavy metal (Cr^6+^) rapid detection kit (HKM). The adsorption capacity (or handling capacity) and the total removal ratios of Cr^6+^ or total Cr were calculated from the following equations:

8 $${q}_{e}=({C}_{0}-{C}_{e})/X$$

9 $$Removalratio( \% )=({C}_{0}-{C}_{e})/{C}_{0}\times 100$$

where q_e_ is the handling capacity of the sorbent, X is the sorbent concentration (g/L), and C_0_ and Ce are the initial and equilibrium concentrations of chromium (Cr or Cr^6+^) (mg/ L), respectively.

Two kinetic models, a pseudo-first-order model and a pseudo-second-order model, were used to characterize the adsorption kinetics of chromium (Cr or Cr^6+^) on the loaded activated carbon composites (NZVI/GAC). The integrated forms of the two models are as follows:

10 $${q}_{t}={q}_{e}[1-\exp (\,-\,{k}_{t})]$$

11 $${q}_{t}=t/[(1/{k}_{2}{{q}_{e}}^{2})+(t/{q}_{e})]$$

where q_e_ is the amount of chromium ions adsorbed at equilibrium (mgg^−1^), and k_1_ (s^−1^) and k_2_ (gmg^−1 s−1^) are the rate constants of the pseudo-first and pseudo-second-order adsorptions, respectively^57^.

The coefficient of determination (R^2^) and the root mean square deviation (RMSD) were used to measure the ability of the models to accurately fit the data. The RMSD was calculated as follows:

12 $$RMSD=\sqrt{\frac{1}{n}{\sum }_{i=1}^{n}{({q}_{m}-{q}_{t})}^{2}}$$

where n is the number of experimental points and q_i_m and q_i_c are the ith measured and calculated values, respectively. A higher R^2^ value and a lower RMSD value indicate a better fit^58^.

To study the equilibrium time and kinetics of Cr^6+^ adsorption by NZVI/GAC, batch experiments were performed under optimized conditions (i.e., 0.2 g/L adsorbent, an initial Cr ion concentration of 118 mgL^−1^ (according to the results of ICP-MS), 30 °C, 100 rpm agitation and pH 6.5) at different time intervals.

Three common isotherm models, i.e., the Langmuir (13) (Langmuir I, 1918), Freundlich (14) (Freundlich HMFU, 1906)^59^, and Langmuir-Freundlich (15) (Sips R, 1948)^60^ models, were used to describe the adsorption equilibrium as follows:

13 $${q}_{e}={K}_{L}{q}_{m}{C}_{e}/(1+{K}_{L}{C}_{e})$$

14 $${q}_{e}={K}_{F}{C}_{e}^{1/n}$$

15 $${q}_{e}={q}_{m}{({K}_{L}^{\text{'}}{C}_{e})}^{n\text{'}}/[{(1+{K}_{L}^{\text{'}}{C}_{e})}^{n\text{'}}]$$

where C_e_ is the residual at equilibrium (mgL^−1^); q_m_ is the maximum adsorption capacity (mgg^−1^); K_L_ is the Langmuir adsorption equilibrium constant (Lmg^−1^), K_F_ and n are the Freundlich constants representing variation of the adsorption capacity (L^−1/n^mg^− (1−n)^g^−1^) and the adsorption intensity with the degree of heterogeneity, respectively; and K_L_′ and n′ are the Langmuir adsorption equilibrium constant (Lmg^−1^) and the adsorption intensity, respectively.

To study the adsorption isotherm, experiments were conducted using different initial Cr^6+^ concentrations ranging from 0 to 175 mg/L. Other conditions were applied as follows: 0.2 g/L NZVI/GAC (new material), 30 °C, 100 rpm agitation, and pH 6.5. For the kinetic experiments, NZVI/GAC were mixed with synthetic water, and the residual chromium (Cr and Cr^6+^) concentration was measured at various time intervals for up to 6 h.

### Stability test of the synthetic NZVI/GAC and the control NZVI

To evaluate the Cr^6+^ removal performance stability of the synthetic materials of NZVI/GAC and their control material NZVI, samples with storages of two months, 12 months and 19 months were used as adsorbents with dosages of 0.2–1.0 g/L and 10.0 g/L to treat Cr^6+^ solutions with concentrations ranging from 0 to 300 mg/L.

The synthetic materials (NZVI/GAC) were synthesized from the same raw materials at different times by the same method. The control material, NZVI, was synthesized from the same raw materials at different times by the same method but without loading GACs. The synthetic material and the control material were placed in a compact bag with nitrogen preservation after vacuum pumping in a desiccator containing desiccant. Cr^6+^ solutions were prepared with pure water and potassium dichromate (analytic reagent grade) at initial concentrations of 15–300 mg/L Cr^6+^. The processing system used a 50-mL Cr^6+^ solution and 10–500 mg of NZVI/GAC, which were mixed and reacted for 24 h at room temperature. At 24 h, the samples were centrifuged at 12600 rpm for 2 min, and the residual concentrations of Cr^6+^ in the solutions were analysed with a heavy metal (Cr^6+^) rapid detection kit. The adsorption capacity (or handling capacity) and the total Cr^6+^ removal ratio were calculated as described above.

### Study of the growth characteristics and growth curves of *Shewanella* MR-1

*Shewanella oneidensis* MR-1 was cultured in LA medium and inoculated into test tubes containing the following liquid media: NB, LB, NX, MRS and TSB (Guan Dong Huankai Microbial Technology Co., Ltd., China). The tubes were cultured at 25–37 °C under aerobic conditions with 150–180 rpm agitation. Growth was determined by observing changes in the turbidity of the fermentation broth.

An automatic analyser (Bioscreen C) with 100-pore plates was used to analyse the growth curves. *S*. *oneidensis* MR-1 was cultured in NB, LB, NX, MRS and TSB media at concentrations of 1%, 5% and 10% with agitation at 150 rpm and 30 °C for 45 h. The MR-1 seed culture process for generating curves was as follows: 150 mL of TSB medium with 5% inoculation in a 500-mL conical bottle cultured for 24 h at 150 rpm and 30 °C.

### Cr^6+^ removal by NZVI/GAC and its synergistic relationship with the MR-1 suspension

NZVI/GAC (2#, new material) at concentrations of 0.2, 0.4, 0.6, 0.8, 1.0, 1.2, 1.8, and 2.0 g/L alone or with MR-1 suspensions of 0.7–2.0% (v/v) were used as the adsorbent with the following adsorbent formula names: I–VIII and IM–VIIIM. The MR-1 suspensions were prepared as follows. First, the MR-1 fermentation broth was prepared as the MR-1 seed, and then wet mud was obtained by centrifuging the fermentation liquid for 10 min at 8500 rpm. Sterile physiological saline was added in a volume equivalent to 1/10 of the fermentation liquid, and the MR-1 bacterial suspension was prepared from the obtained wet mud. The Cr^6+^ solution was prepared with pure water, 37.5 mg/L Cr^6+^, 2 mmol/L sodium formate, 10 mg/L PAC-02 and 0.5 mg/L PAM. The processing system used 50 mL of Cr^6+^ solution and NZVI/GAC with or without the MR-1 suspension, which were mixed and reacted for 23 h at room temperature. Sampling and centrifugation were conducted after 30 min and 23 h to determine the Cr^6+^ concentrations in the supernatant using a Cr^6+^ kit.

### Cr^6+^ removal by the NZVI/GAC detoxification reaction column and the synergistic effects of auxiliary water treatment reagents

Considering the treatment of water sources with nanoiron active carbon material, some unnecessary products, such as different valence states, may exist in the water after the reaction. Cr^6+^ removal and the synergistic effects of the NZVI/GAC detoxification reaction column were tested by adding auxiliary water treatment reagents, such as PAC-02, PAM and sodium formate. First, two identical columns were constructed with 0.5 g of NZVI/GAC (new material), 30 g of K-04 granular activated carbon (Hainan Star Activated Carbon Co., Ltd., model K-04,10–20 mesh), and aqueous hexavalent chromium, with and without auxiliary agents, including 2 mmol/L sodium formate + 10 mg/L PAC-02 + 0.5 mg/L PAM. An initial concentration of 26 mg/L Cr^6+^ (calculated value) was used, and continuous column reactions were carried out at a flow velocity of 4.5–5.0 mL/min. The volume of the inflection point (mL) was measured using a hexavalent chromium rapid detection kit. ICP-MS was used to measure the chromium content at the inflection point and in the outflow water both before and after the inflection point.

### Continuous Cr^6+^ removal from raw water and regeneration of the immobilized reaction column with highly reactive NZVI/GAC using an MR-1 bacterial solution

The Cr^6+^ detoxification reaction columns were glass columns (3.5 cm diameter and 60 cm height) packed to a fixed bed depth of 15.0 cm with 0.5 g of NZVI/GAC (1#), 30 g of K-04 (granular activated carbon for pure water sterilization), approximately 0.5 g of cotton and 5.0 g of macroporous resin soaked in ethanol. A Cr^6+^ solution with an initial concentration of 26 mg/L Cr^6+^ (calculated value) was prepared with raw water and auxiliary agents including 2 mmol/L sodium formate + 10 mg/L PAC-02 + 0.5 mg/L PAM, and continuous column reactions were carried out at a flow velocity of 4.5–5.0 mL/min. Samples were collected from the effluent to measure the residual Cr^6+^ concentration using a Cr^6+^ kit. The MR-1 seed culture processwas as follows: 300 mL of TSB medium with 5% inoculation in a 500-mL conical bottle cultured for 24 h at 150 rpm and 30 °C. After fermentation, the fermentation broth was placed in a refrigerator for preservation for 0–2 weeks, and 2.0 mmol/L sodium formate was added before use.

The maintenance procedures included water washing, pickling and regeneration. When the column was finished, approximately 100 mL of 0.01 mmol/L diluted hydrochloric acid (soaking for 0.5 h) and 200 mL of pure water were used to elute the soaking column with 150 mL of the MR-1 suspension at room temperature (approximately 25–28 °C) for 3 d before use with the Cr^6+^ detoxification reaction columns, which were washed again with approximately 250–300 mL of pure water.

### Characterization

The surface morphologies of the iron-loaded activated carbon composites (NZVI/GAC) prepared in this study were examined by SEM (FESEM, JEOL Ltd., Japan), and the sizes of the NZVI, GAC and NZVI/GAC were obtained by TEM (TEM, JEOL Ltd., Japan). XRD patterns were collected with an Ultima IV instrument at a scan rate of 2 degrees per minute with a 2-h range of 10–90 degrees with Cu K-beta radiation operating at an accelerating voltage of 40 kV to determine the crystal structures and chemical compositions of these particles (1: GAC-BCS5 stored for one year; 2: NZVI stored for two months; 3: NZVI stored for one year; 4: NZVI stored for 19 months and 5: NZVI/GAC stored for two months). FTIR spectra (Perkin-Elmer Instrument Co. Ltd., USA) were collected to analyse the surface chemical structures and compositions of the NZVI, GAC-BCS5 and NZVI/GAC particles. BET surface areas were measured using a surface area analyser (BELSORP-max, MDTC-EQ-M0302) according to GB/T 19587–2004. In addition, the zeta potentials of the powders were measured using a Zetasizer (NaNoZS) according to GB/T 32668-2016, and the total Fe contents of the samples were measured via ICP-MS (Spectro Arcos II, model: MDTC-EQ-M21-01) according to JY/T 015-1996.

### Statistical analysis

Each set of batch sorption experiments was conducted in triplicate. One-way ANOVA with Dunnett’s post hoc test and Tukey’s multiple comparison test was conducted using GraphPad Prism 5.0 software to determine the statistical significance of the results.

## Supplementary information

S1
