# Supplementary material for: Chromium ion removal from raw water by magnetic iron composites and Shewanella oneidensis MR-1
Source: Sci Rep. 2019 Mar 6;9:3687. doi: 10.1038/s41598-018-37470-1 (PMC6403255; doi:10.1038/s41598-018-37470-1)
Supplement: Supplementary file 1 — S1 [file 41598_2018_37470_MOESM1_ESM.docx]

**Chromium ion removal from raw water by magnetic iron composites and *Shewanella oneidensis*** MR-1

Huiqing Wu^1,2^, Qingping Wu^1,2^*, Jumei Zhang, Qihui Gu^1,2^, Linting Wei^1,2^, Weipeng Guo^1,2^, Minhong He^3^

^1^Guangdong Institute of Microbiology, State Key Laboratory of Applied Microbiology Southern China; Guangdong Provincial Key Laboratory of Microbial Culture Collection and Application, Guangdong Open Laboratory of Applied Microbiology, 510070, Guangzhou, P.R. China

^2^Guangzhou Institute of Chemistry, Chinese Academy of Sciences, Guangzhou, 510650, P.R. China

^3^Guangzhou Panyu Zhong Village Tap Water Co., Ltd., Guangzhou, Guangdong, China, 511495

* wuqp203@163.com

**Supplementary materials**

**Cost estimation for powdered activated carbon in the special formulation**

The final mass of the specially formulated powder obtained from an initial dry biomass mixture of 500 g via fermentation, drying (50-90°C) and anaerobic carbonization at 300°C for 2-3 h in a muffle furnace with a 70% maximum heating rate was approximately 180 g (mean value, SD: 5%). The solid-medium formulation of biomass materials was developed from a mixture of corn, wheat bran and soybean meal in a ratio of 3:4:2 by weight. For every 500 g of dry biomass mixture, nutritive salts dissolved in 400 mL of pure water were added, including 0.1% MgSO_4_·7H_2_O, 0.1% peptone, 0.1% KH_2_PO_4_, 0.2% K_2_HPO_4_·3H_2_O, 0.1% FeSO_4_ 7H_2_O, and 0.1% CaCO_3_.

The price of the special formula of activated carbon was estimated based on two considerations: the cost of the material and the processing cost. For the production process of the special formula of activated carbon, the material cost mainly included the biomass materials and some nutrients, and the processing process includes fermentation, drying, grinding and sieving, carbonization and so on. The cost of the biomaterials was calculated on the basis of wholesale prices found on the internet; nutrients were calculated based on the prices of reagents purchased by the unit, and processing costs were calculated on the basis of 10 times the cost of the materials. The material cost calculation basis was as follows: 2.6 yuan/kg for corn, 1.36 yuan/kg for wheat bran, and 3.58 yuan/kg for soybeans, yielding a total of 20.4 yuan for 9 kg of biomass mixture. A total of 7.2 kg of nutritive salts was used, including 7.2 L * (0.1% MgSO4.7 H2O + 0.1% peptone + 0.1% KH_2_PO_4_ + 0.2% K_2_HPO_4_.3 H_2_O + 0.1% FeSO4.7 H2O + 0.1% CaCO3), and the cost of the nutritive salts was 20.64 yuan/kg. The raw material cost of 9 kg of fermentation substrate (raw material) is thus approximately 42 yuan, and 3.2 kg of activated carbon powder can be obtained. Therefore, the material cost of 1 kg of activated carbon powder is 13.2 yuan, and using a processing cost 10 times that number, the total price is approximately 100 yuan per kg of GAC.

**Cost estimation for NZVI/GACs**

The preparation costs included material costs and processing costs: the preparation of 2500 g of NZVI/GAC materials required 1 kg of GAC + 100 g of PEG-6000 + 4.45 kg of FeSO4.7H2O (Guangzhou Jardine Reagent 23.0 yuan/kg) + approximately 880 g of KBH4 (MW=53.94, 120 yuan/kg) or 615 g of NaBH4 (MW=38.3, 1160 yuan/kg reagent level, the amount of KBH4 in the iron loading process can be optimized to reduce the molar ratio of Fe^2+^ to 1:1 or 1:1.05.) + 3000 ml of anhydrous ethanol (6.8 yuan/kg). The processing steps included ultrasonication and reaction, centrifugation, vacuum drying, crushing and sieving. The cost of reducing the NZVI/GACs with potassium borohydride was 115 yuan/kg of material. With a processing cost of 100 yuan/kg, the total price was approximately 197.5 yuan/kg. The most expensive material was reagent-grade KBH_4_, which had a wholesale price of 120 yuan/kg. Analytical-grade FeSO4.7H_2_O cost 23.0 yuan/kg. The cost of rigid NZVI/GACs was further reduced by using bulk KBH4 and ferrous citrate (350 yuan/ton) at 85 yuan/kg of material. Batch production reduced the processing cost per unit weight of the product to 100 yuan/kg, and thus the total price of NZVI/GACs was approximately 180 yuan/kg. Overall, the estimated cost of NZVI/GACs is not more than 200 yuan per kg.

**Initial purification process of graphene from high-purity graphite**

The main process in the preparation of graphene was milling high-purity graphite at 500 rpm for 45 min under low temperature (- 20 ℃, where polyethylene glycol was used as antifreeze) by adding methanol and then heating the paste under vacuum. Because a larger machine (12 L) was used, only 480 grams of dry sample was obtained from 1 kg of high-purity graphene.

One hundred and thirty grams of ball-milled high-purity graphite dry powder was added to 1300 mL of DMF solvent. The whole purification process referred to Yan Liu (2108). The ball-milled mixture dissolved in DMF solvent was shaken with ultrasonic waves for 2 min, followed by centrifugation at 500 rpm for 15 min. The upper liquid was centrifuged at 11000 rpm for 10 min, and the recovered DMF solvent was used repeatedly. The precipitate obtained at 500 rpm was dissolved in the recovered DMF solvent and centrifuged again at 500 rpm for 15 min. This process was repeated 6 times. The precipitate obtained at 11000 rpm was then washed twice with ultrapure water and centrifuged at 11000 rpm for 10 min. After vacuum-drying the washed 11000-rpm precipitate, 50.32 g of preliminarily purified graphene dry product was obtained as the initially purified graphene.

Reference: Yan Liu ，et al. A facile strategy for preparation of magnetic graphene oxide composites and their potential for environmental adsorption. *Ceramics International* (2018). Https: //doi.org/10.1016/J.ceramint.2018.07.081.

**Synthesis of nanoiron composites loaded with different carbon materials**

Preparation began with 10 g of 100-mesh carbon carrier and 1.6 mol/L FeSO_4_·7H_2_O in 100 mL of 1% PEG-6000 with degassed water treated with ultrasound at 80 kHz for 120 min. Subsequently, a freshly prepared NaBH_4_ solution in 30% (v/v) degassed water/absolute ethanol (moles of NaBH_4_:Fe^2+^=2:1) was added to the mixture at a rate of 50–60 drops per minute. After delivering all of the NaBH_4_ solution (dissolved in degassed water), the mixture was sealed for 2 h of quiescent reaction. Next, the prepared composites were separated from the solution by centrifugation, followed by rinsing with deionized water and centrifugation twice. The composites were then dried under vacuum in a drying oven at 80–90°C and stored in vials for subsequent experiments under protection by inert argon gas. A mean of 25.0 g of nanoiron active carbon composites (SD: 5%) was obtained from 10 g of carbon carrier (including GAC BCS5, the preliminary purification of graphene (PPG) and high-purity graphite (HPG), all as 100 mesh (Table S1)).

Table S1 Yield of nanoiron composites from 3 different carbon materials

| No. of sample | Wt (g) | Loading material | Synthetic material |
| --- | --- | --- | --- |
| 7# | 25.8 | GAC BCS5 | Fe @GAC |
| 8# | 23.39 | PPG | Fe@ PPG |
| 9# | 22.91 | HPG | Fe@ HPG |

Note: Numbers of samples in this paper: 7#, NZVI/GACs (2018091802); 8#, NZVI/Graphene (2018091804); 9#, NZVI/HPG (2018091806). All studies were conducted within the validity period (not exceeding one year, usually within two months) except for the validity test samples.

**Performance evaluation of nanoiron composites with different loads**

To evaluate the superiority of the activated carbon materials loaded with nanoiron, three different materials were used as binders to synthesize nanoiron-loaded composite materials: activated carbon powder with the special formula (GAC BCS5), the initially purified graphene and the high-purity graphite powder. The three materials (Fe@GAC, Fe@ PPG, and Fe@ HPG) were synthesized by the same nanoiron process, and the adsorption capacities of the three materials for removing hexavalent chromium from water were tested. The concentration of the adsorbent was 1 mg/L, and the initial concentration of hexavalent chromium in the water was 140.22 mg/L. After standing for 17 h, the samples were centrifuged at 12000 rpm for 2 min, and the content of chromium in the supernatant was determined by ICP-MS. The results are shown in Table S2.

Table S2 Cr^6+^removal from water by nanoiron composites with different loads

| Sample | Ce(Cr^6+^, mg/L） | R% | Qe(mg/g) |
| --- | --- | --- | --- |
| 7# | 60.62 | 56.68 | 79.48 |
| 8# | 66.64 | 52.39 | 73.46 |
| 9# | 53.61 | 61.67 | 86.47 |
| CK | 140.22 | 140.22 | 140.22 |

The results showed that when the initial concentration of hexavalent chromium in the water was 140.22 mg/L, the adsorption capacities (mg of Cr/g of material) of the three materials (Fe@GAC, Fe@ Graphene，Fe@ Graphite) were considerable. In addition, the price of high-purity graphite is 200–300 yuan per kg, but the cost of homemade powdered inactive carbon materials and the nanoiron composites **(**NZVI/GACs) is approximately 100 and 200 yuan per kg, and the cost performance ratio of the GAC BCS5 is higher than those of high-purity graphite materials, Fe@ Graphite and Fe@ Graphene.

Reference

1. Ahmad Amiri, et al. Facile, environmentally friendly, cost effective and scalable production of few-layered graphene. *Chemical Engineering Journal* **326**, 1105–1115(2017).

**Evidence for regeneration by the MR-1 suspension**

The Cr^6+^ detoxification reaction columns (A) were packed to a fixed bed depth of 15.0 cm with 0.5 g of NZVI/GACs and 30 g of K-04 (granular activated carbon for pure water sterilization) in glass columns with a diameter of 3.5 cm and a height of 60 cm with approximately 0.5 g of cotton and 5.0 g of macroporous resin soaked in ethanol. A Cr^6+^ solution with an initial concentration of 26 mg/L Cr^6+^ (calculated value) was prepared with raw water and auxiliary agents including 2 mmol/L sodium formate + 10 mg/L PAC-02 + 0.5 mg/L PAM, and continuous column reactions were carried out at a flow velocity of 4.5–5.0 mL/min. Samples were collected from the effluent to measure the residual Cr^6+^ concentration using a Cr^6+^ kit. For the MR-1 seed culture process for generating curves, 300 mL of TSB medium was inoculated with a 5% inoculum in a 500-mL conical bottle and cultured for 24 h at 150 rpm and 30°C. After fermentation, the fermentation broth was placed in a refrigerator for preservation for 0–2 weeks; 2.0 mmol/L sodium formate was added before use.

Maintenance procedure 1 included water washing and regeneration; when the column was finished, approximately 200 mL of pure water was used to elute the soaking column with 150 mL of the MR-1 suspension at room temperature (approximately 25–28°C) for 3 d before use with the Cr^6+^ detoxification reaction columns, which were washed again with approximately 250–300 mL of pure water. Maintenance procedure 2 included water washing, pickling and regeneration; before washing and regeneration, approximately 100 mL of 0.01 mmol/L dilute hydrochloric acid (soaking for 0.5 h) was added; the remaining steps were the same as in maintenance procedure 1.

The effect of the regeneration column on the removal of hexavalent chromium from water was indicated by the volume of the inflection point and the saturation point. The inflection point (IP) for Cr^6+^ was observed at approximately 0.025 mg/L and Ct/C_0_=0.001; the saturation point (SP) was Ct/Co=0.8–0.9. The regenerated column after washing regeneration procedure 1 was called A1-1, the regenerated column after washing regeneration procedure 2 was called A2-1, and the regenerated column after washing regeneration procedure 2 was used to maintain the column twice was called A2-2. The results are shown in Table S3.

Table S3 Cr^6+^ removal effect of the regenerated columns

| Running program | A | | A1-1 | | A2-1 | | A2-2 | |
| --- | --- | --- | --- | --- | --- | --- | --- | --- |
| Key point | IP | SP | IP | SP | IP | SP | IP | SP |
| V(ml) | 850 | 2917 | 750 | 2415 | 800 | 2515 | 700 | 2335 |

The results showed that soaking in 0.01 N HCL for half an hour and soaking with 150 mL of MR-1 fermentation broth at room temperature for 3 days were superior to soaking with 150 mL of fermentation broth alone at room temperature for 3 days. In addition, the MR-1 bacteria played a major role in the regeneration of system reaction activity, and thus, MR-1 fermentation broth could be used as a regeneration agent.
